# Assessing the Efficacy of Current Histopathological Tumor Reporting Systems for Evaluating the Response to Neoadjuvant Chemotherapy for Breast Carcinoma: Protocol for an Observational Study

**DOI:** 10.2196/56825

**Published:** 2025-06-16

**Authors:** Anita Sajjanar, Sunita Vagha

**Affiliations:** 1 Department of Pathology Datta Meghe Medical College Shalinitai Meghe Hospital and Research Center Nagpur India; 2 Jawaharlal Nehru Medical College Sawangi India

**Keywords:** evaluation, comparison, histopathological assessment systems, breast carcinoma, neoadjuvant chemotherapy, prognosis, hormone receptor status, lymphnode status, residual cancer burden, Miller-Payne system, Chevallier classification, Sataloff classification, AJCC residual tumor size (R), clinical decision making, treatment outcome

## Abstract

**Background:**

With a predicted 2 million new cases identified globally in 2018, breast carcinomas are the most common cancer in women and the primary cause of cancer-associated mortality. In the management of breast cancer, neoadjuvant chemotherapy treatment (NACT) has become a mainstay, particularly for patients with inflammatory and locally advanced breast cancer. It increases the possibility of breast-conserving surgery, facilitates tumor downstaging, and gives early indications of the effectiveness of treatment. Evaluating the histopathological response after NACT is crucial for prognosis and guiding subsequent treatment decisions. This study explores the various histopathological assessment systems used in breast carcinoma patients after NACT, focusing on the residual cancer burden (RCB) score, Miller-Payne system, Chevallier classification, Sataloff classification, National Surgical Adjuvant Breast and Bowel Project (NSABP) Protocol B-18 system, and the American Joint Committee on Cancer residual tumor size (R) categories. We compare their methodologies, strengths, limitations, and clinical significance, providing a detailed analysis of their roles in improving patient outcomes.

**Objective:**

The aim of this study is to confirm the diagnosis of breast carcinoma based on histopathology, to evaluate various scoring systems through assessments of histomorphological features affecting post-NACT patients with breast carcinoma, to compare the various systems regarding the response to therapy and forming prognoses, and to develop an ideal histomorphological assessment system for breast carcinoma in post-NACT patients. The study also focused on how breast tumors respond to NACT and how this response can guide treatment decisions and improve the formulation of prognoses.

**Methods:**

This observational study will be retrospective and prospective; it will include 128 patients diagnosed with breast carcinomas who have undergone NACT and were referred to a tertiary care hospital between January 2019 and December 2024. Following chemotherapy, a thorough examination of the histopathological specimens will be conducted to assess any changes in histomorphology.

**Results:**

Data collection started in September 2021 and will be completed by December 2025. Data analysis began in January 2025, and the results are expected to be published in December 2025. Institutional ethics committee clearance was obtained prior to commencement of the study. This is a nonfunded academic study.

**Conclusions:**

This project aims to evaluate and compare histopathological assessment systems in patients with breast carcinoma after NACT. Various histopathological systems, such as the RCB score, the Miller-Payne grading system, and other systems, each provide valuable insights into how well tumors respond to chemotherapy. The aim is to reveal essential histopathological parameters, leading to the refinement and potential modification of grading systems to improve clinical decision-making, treatment outcomes, and personalized care; however, challenges persist in standardization and consensus.

**International Registered Report Identifier (IRRID):**

DERR1-10.2196/56825

## Introduction

### Background

With a predicted 2 million new cases identified globally in 2018, breast carcinomas are the most common cancer in women and the primary cause of cancer-associated mortality [1]. Breast cancer is not a single disease; each tumor has distinct features, such as size, kind, lymph node status, hormone receptor status, and human epidermal growth factor receptor 2 (HER2)/neu oncogene expression. Breast cancer takes diverse forms in different ethnic groups. The multitude of genetic mechanisms that ultimately determine the kind of breast cancer and its underlying biology most likely reflect this variability.

Neoadjuvant chemotherapy treatment (NACT) is used for inflammatory breast cancer and locally advanced breast cancer, as well as to reduce large tumors to allow for breast conservation therapy [2].

NACT has the ability to reduce or even completely reverse primary cancers and their metastases. Patients with clinically node-negative breast cancer who have unfavorable tumor characteristics and are expected to benefit from adjuvant systemic therapy can now also benefit from NACT [3]. NACT offers a number of advantages. With the full pathologic response serving as a proxy predictor of survival, it presents a rare chance to assess the response to therapy, evaluate new therapeutic agents more quickly, and stop ineffective treatments early.

Additionally, patients are spared the burden of toxicity and side effects when they switch to a different medication or adjust the dose if they become resistant to therapy. Moreover, NACT permits the collection of tumor samples prior to, during, and following treatment, and offers the chance for tailored therapy [4,5].

In the management of breast cancer, NACT has become a mainstay, particularly for patients with inflammatory and locally advanced breast cancer. It increases the possibility of breast-conserving surgery, facilitates tumor downstaging, and gives early indications of the effectiveness of treatment. Evaluating the histopathological response after NACT is crucial for prognosis and guiding subsequent treatment decisions. This study explores the various histopathological assessment systems used in patients with breast carcinoma after NACT, focusing on the residual cancer burden (RCB) score, Miller-Payne system, Chevallier classification, Sataloff classification, National Surgical Adjuvant Breast and Bowel Project (NSABP) Protocol B-18 system, and the American Joint Committee on Cancer (AJCC) residual tumor size (R) categories.

The RCB score is the system recommended in the European standards for assessing nodal and tumor regression. Three reactions are categorized under the NSABP B-18 system. The Miller-Payne system incorporates the change in cellularity between biopsy and resection specimens, while the Sataloff and RCB systems take into account the condition of the lymph nodes and residual tumor, and the Chevallier grade takes into account the possibility of some regression.

This research has four main aims: (1) individually assess various parameters used in different grading systems for breast carcinoma for making treatment prognoses after the chemotherapeutic intervention; (2) evaluate and juxtapose the grading systems; (3) determine the effectiveness of individual parameters for various grading systems so as to better understand treatment outcomes in patients and help clinicians assess ongoing treatment modalities and modulate them if required; (4) improve the reliability of prognosis prediction through better evaluation.

### Review of Literature

Residual in situ carcinomas have imprecise prognostic signiﬁcance, which was taken into account by Chevallier et al [6] in the pathological classiﬁcation system they reported in 1993. In 1995, the breast and axillary nodes were examined pathologically for therapeutic effects by Sataloff et al [7], who developed a grading scale. The NSABP B-18 trial by Fisher et al [8] in 1997 and 1998 was one of the largest studies comparing neoadjuvant to adjuvant therapy. In 2003, a new histological grading system to assess the response of breast cancers to primary chemotherapy, the Miller-Payne system, was introduced that did not include the assessment of axillary lymph nodes; it assessed prognostic significance and survival [9].

The RCB score was developed by Symmans and coworkers [10] in 2007, and it is highly recommended that it be included in routine histopathological reports of breast cancers treated with NACT. While the majority of RCB features are commonly included in histopathological reports, certain elements, such as the proportion of ductal carcinoma in situ, cellularity, and the second greatest dimension of tumor size, need to be characterized by experience in the field. Standardized methods for reporting these markers are included in concise guidance at the RCB calculator website [11].

### Knowledge Gap Analysis

**Figure 1:**
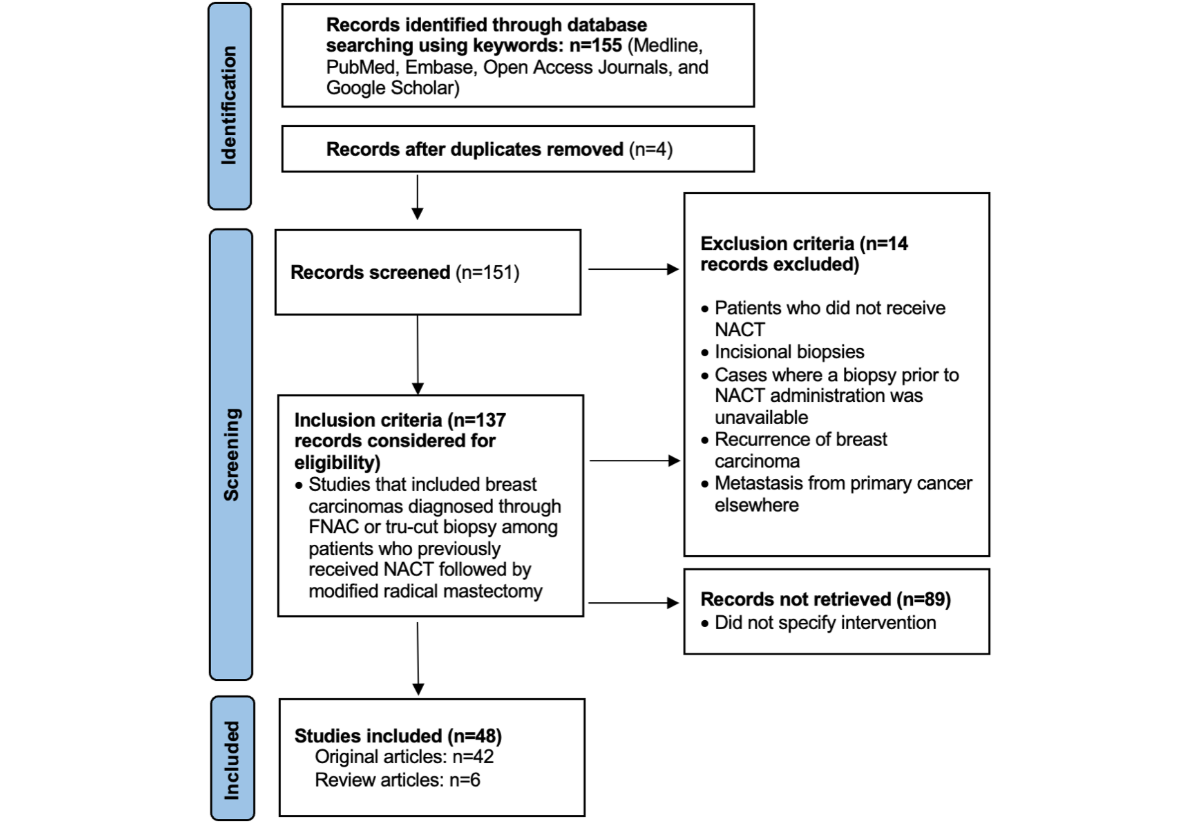
PRISMA (Preferred Reporting Items for Systematic Reviews and Meta-Analyses) flowchart. FNAC: fine needle aspiration cytology; NACT: neoadjuvant chemotherapy treatment.

Many evaluation systems are presently in vogue for the assessment of histomorphological changes in post-NACT patients with breast carcinoma. Systems used to assess changes after therapy include the Chevallier grading system, Miller-Payne grading system, Sataloff grading system, and RCB score. These assessment systems have varying parameters to evaluate histomorphological changes. Because there are multiple assessment systems, assessments can vary indirectly, affecting post-NACT sequalae in patients. There is no international agreement on terminology, and how regression is defined varies from nation to nation. Hence, it is necessary to determine fixed criteria or a single system for the assessment of post-NACT patients. Despite the introduction of a number of national guidelines in Australia, Belgium, Germany, the United Kingdom, the Netherlands, the United States, and Hungary, among other countries, with the goal of standardizing specimen collection and reporting, there is no worldwide consensus on the interpretation of tumor regression, the definition of a pathologic complete response, or the measurement of tumor size in cases where fibrosis develops as a result of NACT or there is multifocality. Figure 1 shows the PRISMA (Preferred Reporting Items for Systematic reviews and Meta-Analyses) flow chart of the results, Textbox 1 shows the keywords used in the literature review, and Table 1 provides an overview of the various grading systems.

A comprehensive review of the literature was conducted, encompassing studies that used various grading systems for assessing histomorphological changes in patients with breast carcinoma undergoing NACT. The selected grading systems, including NSABP B-18, Chevallier, Sataloff, Miller-Payne, and RCB, were analyzed in terms of their parameters, methodologies, and reported prognostic implications.

**Keywords and translations used in the literature search.**

**Keywords**

(“histopathologic” [All Fields] OR “histopathological” [All Fields] OR “histopathologically” [All Fields]) AND (“assess” [All Fields] OR “assessed” [All Fields] OR “assessment” [All Fields] OR “assesses” [All Fields] OR “assessing” [All Fields] OR “assessment” [All Fields] OR “assessment s” [All Fields] OR “assessments” [All Fields]) AND (“system” [All Fields] OR “system s” [All Fields] OR “systems” [All Fields]) AND ((“breast neoplasms” [MeSH Terms] OR (“breast” [All Fields] AND “neoplasms” [All Fields]) OR “breast neoplasms” [All Fields] OR (“breast” [All Fields] AND “carcinoma” [All Fields]) OR “breast carcinoma” [All Fields]) AND (“patient s” [All Fields] OR “patients” [MeSH Terms] OR “patients” [All Fields] OR “patient” [All Fields] OR “patients s” [All Fields])) AND (“neoadjuvant therapy” [MeSH Terms] OR (“neoadjuvant” [All Fields] AND “therapy” [All Fields]) OR “neoadjuvant therapy” [All Fields] OR (“neoadjuvant” [All Fields] AND “chemotherapy” [All Fields]) OR “neoadjuvant chemotherapy” [All Fields])

**Translations**

- Histopathological: “histopathologic” [All Fields] OR “histopathological” [All Fields] OR “histopathologically” [All Fields]
- Assessment: “assess” [All Fields] OR “assessed” [All Fields] OR “assessment” [All Fields] OR “assesses” [All Fields] OR “assessing” [All Fields] OR “assessment” [All Fields] OR “assessment’s” [All Fields] OR “assessments” [All Fields]
- Systems: “system” [All Fields] OR “system’s” [All Fields] OR “systems” [All Fields]
- Breast carcinoma: “breast neoplasms” [MeSH Terms] OR (“breast” [All Fields] AND “neoplasms” [All Fields]) OR “breast neoplasms” [All Fields] OR (“breast” [All Fields] AND “carcinoma” [All Fields]) OR “breast carcinoma” [All Fields]
- patients: “patient’s” [All Fields] OR “patients” [MeSH Terms] OR “patients” [All Fields] OR “patient” [All Fields] OR “patients’s” [All Fields]
- Neoadjuvant chemotherapy: “neoadjuvant therapy” [MeSH Terms] OR (“neoadjuvant” [All Fields] AND “therapy” [All Fields]) OR “neoadjuvant therapy” [All Fields] OR (“neoadjuvant” [All Fields] AND “chemotherapy” [All Fields]) OR “neoadjuvant chemotherapy” [All Fields]

**Table 1:** Various grading systems.

System	Report	Patients, n	Pathological complete response, %	Validated as prognostic for survival	Parameters included	Limitations
Chevallier method	Chevallier et al [6], 1993	45	26	OS^a^, DFS^b^	Tumor cells, ductal carcinoma in situ, and lymph nodes	Does not assess tumor cellularity, LVI^c^, or tumor heterogenicity
Sataloff method	Sataloff et al [7], 1995	36	16	DRFS^d^	Therapeutic effect on tumor cells and lymph nodes	This system does not include tumor cellularity, LVI, or tumor heterogenicity
NSABP B-18^e^	Fisher et al [8], 1998	1234	20	OS, DFS	Presence of invasive carcinoma	Metastases to lymph nodes were analyzed separately; does not include LVI or tumor heterogenicity
Miller-Payne system	Ogston et al [9], 2003	176	14	OS, DFS	Presence of invasive carcinoma tumor cellularity	Does not include the response in lymph nodes or LVI^c^
AJCC^f^	Carey et al, [12] 2005	132	17	OS, DFS	Tumor lymph node status (number and size)	This system does not include changes in cellularity or LVI
RCB^g^ system	Symmans et al [10], 2007	432	24	DRFS	Size of tumor bed, tumor cellularity, lymph node status	This system does not include LVI

^a^OS: overall survival.

^b^DFS: disease-free survival.

^c^LVI: lymphovascular invasion.

^d^DRFS: distant recurrence–free survival

^e^NSABP B-18: National Surgical Adjuvant Breast and Bowel Project Protocol B-18.

^f^AJCC: American Joint Committee on Cancer.

^g^RCB: residual cancer burden.

## Methods

### Study Design

This observational study will be retrospective and prospective and will include 128 patients who were diagnosed with breast carcinomas and underwent NACT after referral to a tertiary care hospital between January 2019 and December 2024.

### Breast Specimens

**Figure 2:**
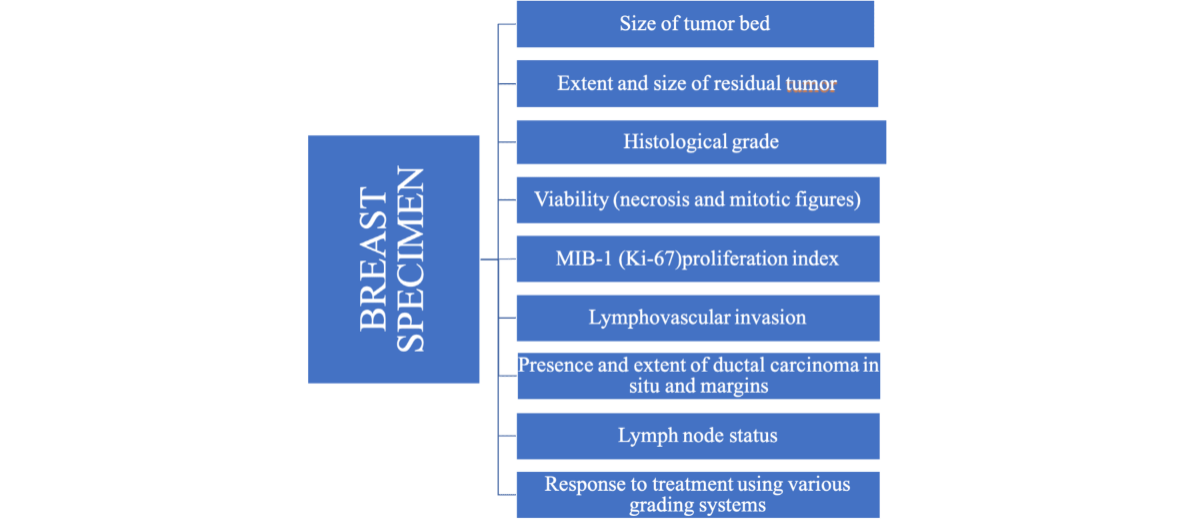
Macro- and microscopic reporting of breast carcinomas.

Histopathological reporting will be performed according to the techniques shown in Figure 2. The pathological reports on breast specimens will include (1) the presence and size of the tumor bed, which is important for documentation, especially in cases with pathologic complete response; (2) the size and extent of residual tumor, including 2D measurements of the largest area of invasive cancer, the number of foci, or number of blocks with foci of invasion; (3) the average cancer cellularity of the residual tumor bed; (4) the appearance of the residual tumor and ita grade, if applicable, and a comparison to pretreatment carcinoma, if possible; (5) viability (necrosis and mitotic figures) and MIB-1 (Ki-67) proliferation index; (6) lymphovascular invasion; (7) presence and extent of ductal carcinoma in situ; (8) margins with respect to tumor bed, as well as invasive and in situ carcinoma. The report will also include a comment on the overall response to treatment and the lymph node status.

### Assessment of Pathological Tumor Response

Histopathological evidence on the chemotherapeutic response will be graded based on hematoxylin and eosin (H&E)-stained sections on the basis of the parameters used by various histopathological assessment systems for patients with breast carcinoma after NACT.

### General Methodology

A detailed history will be obtained for each patient, and a clinical examination will be performed. Radiological investigations and previous biopsies will be noted from the medical record of each patient. The resected specimens obtained after modified radical mastectomy (MRM) following NACT will be studied as per standard protocol. All specimens are fixed in 10% formalin and grossed; sections are taken from the lesion and the lymph nodes. The samples are processed and embedded in paraffin. The H&E-stained paraffin sections are then studied under the microscope. The histopathological specimens collected after chemotherapy were evaluated for histomorphological changes.

### Statistical Considerations

Continuous variables will be summarized using tables of descriptive statistics: number of patients with recorded observations, mean, SD, median, minimum, and maximum. Categorical variables will be summarized using counts and percentages. Descriptive statistics will be presented by diagnosis. Diagnostic outcomes will be tabulated and compared for a unified post-NACT tumor reporting system.

### Analytical Procedures

All results will be analyzed using Stata (version 15; StataCorp). We will calculate the sensitivity, specificity, positive predictive values, and negative predictive values for modification of the existing grading systems. Agreement analysis (primary and secondary end points) will be run to determine the percentage of agreement (overall, positive, and negative agreement) between both procedures, as well as κ coefficients, *P* values, and 95% CIs.

Continuous variables will be summarized using descriptive statistics, specifically the mean, SD, median, minimum, and maximum. Comparison of continuous parameters between pairs of groups will be performed using the *t* test or Wilcoxon Mann-Whitney *U* test; paired data will use the Wilcoxon signed rank test (nonparametric), as appropriate. Categorical variables will be summarized using frequencies and percentages, and they will be compared between pairs of groups using the *χ*^2^ or Fisher exact test or, as appropriate, the McNemar *χ*^2^ test for paired data.

### Sample Size

We will include 109 participants; this sample size was calculated using Epi Info (version 7.0; US Centers for Disease Control and Prevention) with a 5% precision level, 95% confidence level, and a design effect of 1%.

### Inclusion and Exclusion Criteria

All patients with breast carcinomas diagnosed by fine needle aspiration cytology or tru-cut biopsy who had previously received NACT followed by MRM will be considered for the study. Patients who did not receive NACT, who underwent incisional biopsies, for whom a biopsy prior to NACT administration was unavailable, or who had recurrence of breast carcinoma and metastasis from a primary cancer elsewhere will not be included.

### Ethics Approval

Institutional ethics committee clearance was obtained prior to commencement of the study (IEC DMIMS [DU]–DMIMS [DU]/IEC/2021/454, dated July 31, 2021). All the data captured were anonymized. A thorough history including clinical examinations, radiological tests, and prior biopsies will be documented from the patient’s medical records in each case. An informed consent form will be provided to the participants enrolled in the study. This is an academic study and no compensation will be given to participants.

### Study Plan

Table 2 shows an outline of the study plan.

**Table 2:** Quarterly scheduling of research work.

Item	Quarter											
	1	2	3	4	5	6	7	8	9	10	11	12
Outline of study design and preparation	✓	✓										
Reference collection and review of literature			✓	✓								
Data collection					✓	✓	✓					
Data compilation and tabulation								✓				
Data analysis									✓			
Thesis writing									✓	✓	✓	
Final thesis preparation										✓	✓	
Submission												✓

## Results

Data collection started in September 2021 and will be completed by December 2025. As of December 2024, a total of 128 patients were recruited who had been diagnosed with breast carcinomas and had undergone NACT at a tertiary care hospital. Data analysis began in January 2025, and the results are expected to be published in December 2025.

## Discussion

### Overview

This study aims to evaluate various scoring systems through assessment of histomorphological features affecting post-NACT patients with breast carcinoma to compare various systems regarding the response to therapy and forming prognoses in order to develop an ideal histomorphological assessment system for breast carcinoma in post-NACT patients. We hypothesize that these assessments focus on how the response to therapy can guide treatment decisions and improve the formulation of prognoses.

### Principal Findings and Comparison to Prior Work

After the literature search, we observed that there is no single reporting system for post-NACT mastectomy specimens that includes all parameters and predictors for management and prognosis of breast carcinoma in patients. The first objective is to confirm the diagnosis of breast carcinoma based on histopathology. The second objective is to evaluate various scoring systems through assessments of histomorphological features affecting post-NACT patients with breast carcinoma. The third objective aims, after comparing the various grading systems for reporting on post-NACT mastectomy specimens and mixed grading systems for breast carcinoma, to develop a more evolved and advanced grading system by modification of the existing systems and incorporation of their chief parameters. The fourth objective aims to help determine the most appropriate treatment protocol, predict treatment outcomes, and accurately speculate on the prognosis of the patient.

### Strengths and Limitations

This study has several strengths. The first is that the study involves the evaluation and comparison of various histopathological assessment systems in post-NACT patients with breast carcinoma. This implies a comprehensive and multidimensional approach to understanding the impact of different evaluation methods on outcomes after chemotherapy.

Second, our focus on post-NACT patients with breast carcinoma indicates a patient-centric perspective, considering the specific challenges and responses in this subgroup. This approach acknowledges the unique nature of breast cancer and the significance of NACT. The study explicitly incorporates various grading systems, such as NSABP B-18, Chevallier, Sataloff, Miller-Payne, and RCB. This reflects a thorough exploration and integration of existing systems, aiming to provide a comprehensive understanding of their respective contributions. The inclusion of keywords like “treatment prognostication,” “tumor response,” and “clinical outcome” reflects a strong emphasis on assessing the practical implications of histopathological assessments. This aligns with the clinical relevance of these systems in guiding treatment decisions and predicting patient outcomes. Keywords like “hormone receptor status,” “HER2/neu,” and “molecular subtypes” indicate an awareness of the diverse molecular characteristics of breast cancer. This reflects an intention to explore how these factors may influence the effectiveness of NACT and subsequent histopathological assessments. The inclusion of “histomorphological changes” emphasizes the examination of structural and morphological alterations in breast carcinomas after NACT. This is crucial for understanding the impact of treatments at a microscopic level.

Overall, the study is geared toward unraveling the clinical implications of using various histopathological assessment systems in post-NACT patients with breast carcinoma. This aligns with the broader goal of improving treatment strategies and patient outcomes in this specific context.

Some limitations of our study include a relatively small sample size, which could affect the generalizability of the findings to a broader population. The study is conducted in a single-center setting, which may limit the diversity of the study population. Differences in interpretation can lead to variability in the assessment of the tumor response. The current tumor reporting system may not adequately predict long-term survival or recurrence in all cases, as it often focuses on the immediate pathologic response without integrating other biological markers. Developing and implementing a system that integrates multiple types of data requires significant adjustments in pathology workflows and clinical practices. Comprehensive reporting requires advanced technology, specialized training, and additional time for analysis, which may not be feasible for all institutions.

### Conclusions

In summary, this project aims to evaluate and compare histopathological assessment systems in post-NACT patients with breast carcinoma because various histopathological systems, such as the RCB score, the Miller-Payne grading system, and other systems, each provide valuable insights into how well tumors respond to chemotherapy. The study aims to understand the essence of histopathological parameters, leading to the refinement and potential modification of grading systems to improve clinical decision-making, treatment outcomes, and personalized care, given that challenges persist in standardization and consensus.

## Abbreviations

AJCC: American Joint Committee on Cancer
H&E: hematoxylin and eosin
HER2: human epidermal growth factor receptor 2
MRM: modified radical mastectomy
NACT: neoadjuvant chemotherapy treatment
NSABP: National Surgical Adjuvant Breast and Bowel Project
RCB: residual cancer burden
